# Anti-*Toxocara* Seropositivity in the Patients with Eye Diseases in Ophthalmology Clinics in Tehran, Iran

**Published:** 2019

**Authors:** Soodabeh EINIPOUR, Farid TAHVILDAR BIDEROUNI, Alireza RAMEZANI

**Affiliations:** 1. Department of Parasitology and Mycology, School of Medicine, Shahid Beheshti University of Medical Sciences, Tehran, Iran; 2. Department of Ophthalmology, Imam Hosein Hospital, Shahid Beheshti University of Medical Sciences, Tehran, Iran

**Keywords:** Uveitis, ELISA, Eye diseases, Toxocariasis, Iran

## Abstract

**Background:**

Eye diseases, including, Uveitis, inflammation of the retina, and choroid are caused by different agents in humans and has a variety of anterior medial and posterior types. The agent of eye diseases is very different from simple bacterial to acute viral, fungal and parasitic infection. There is limited information regarding the type of eye diseases and *Toxocara canis*.

**Methods:**

Blood samples of 359 individuals (339 patients including endophthalmitis, uveitis, DCR, glaucoma, and cataract 20 individuals as control group) together with their information were collected from ophthalmology hospitals in Tehran, Iran from Feb 2013 to Jan 2015. The patient's serum was evaluated for the presence of anti-Toxocara antibodies by ELISA kits and blood smears for high eosinophilia. The positive samples were confirmed by Western blot analysis for *T. canis* infection.

**Results:**

Overall, 339 patients sera with eye diseases and control group were tested for anti-*T. canis* antibodies. Nineteen (5.6%) patients had anti-*T. canis* IgG that 14 (6.1%) were male and 5 (4.5%) were female, and all the patients had negative eosinophilia as well. The results of Western blot analysis for 19 positive patients indicated that 15 were infected by *T. canis* and 4 were infected by other parasitic infection. The results for control group were negative.

**Conclusion:**

All the patients with inflammatory eye diseases such as endophthalmitis, uveitis DCR, glaucoma and cataract were studied for *Toxocara* infection in this research work were at risk. Therefore, in contrast to the previous idea, all eye inflammatory diseases in ocular patients should be considered for *Toxocara* antibodies in addition to Uveitis.

## Introduction

Eye disease is one of the most important problems of human and can cause irreversible harm like blindness. There are so many causes of blindness, but uveitis is one of the major cause. Uveitis is a general term used for inflammation of the uvea. Uveitis can be unilateral or bilateral, Granulomatous or non-granulomatous, Acute anterior uveitis, Intermediate uveitis, Posterior uveitis, and Uveitis-complications has included Glaucoma, Hypotonia, Cataract, Eye Toxoplasmosis, Histoplasmosis and eye toxocariasis (1,2).

### Ocular toxocariasis

Toxocariasis is caused by the second stage larva of *Toxocara canis* and *Toxocara cati,* the signs and symptoms of covert toxocariasis are coughing, fever, abdominal pain, headaches, and changes in behavior and ability to sleep. Upon medical examination, wheezing, hepatomegaly, and lymphadenitis are often noted. High parasitic loads or repeated infection can lead to visceral larva migrans. it is generally a unilateral sickness, which usually shows as retinal granuloma, with a yellowish or whitish inflaming mass, in the rear pole or surrounding retina (1–3). Toxocara antibody titer has considered for VLM and OLM in human in Iran. The prevalence of VLM has reported from 1%–34.5% and OLM from 1%–6.2% in human, but there are just 3 reports about OLM (4). Some of patients with anterior, intermediate and posterior uveitis had positive antibody titer but the other types of eye inflammation were not considered (5).

In the developing countries, parasitic infections such as *Toxoplasma* and *Toxocara* are considered as a major problem, but in underdeveloped countries, less attention has been paid. Otherwise, the ophthalmologists encounter to some uveitis with unknown agent, so using molecular technique helps to find out the correlation between the uveitis and ocular *Toxocara* and *Toxoplasma* infection, so that it seems working on prevalence of uveitis, related to *Toxocara* and *Toxoplasma* is worthy (6–8).

In this study, the prevalence of anti-*T. canis* antibody in patients with eye diseases was investigated by detecting serum antibody and confirmed by Western blot analysis.

## Materials and Methods

In this descriptive study, 339 patients with eye diseases including 229 males and 110 females with idiopathic uveitis symptoms: papilitis, vitritis, endophethalimitis and granuloma, and 20 individuals: 10 males and 10 females without eye disease as control group, were enrolled from Feb 2013 to Jan 2015 in five ophtalemic hospitals in Tehran, Iran. Blood samples were collected from all the individuals to analyze complete blood cell count (CBC), including eosinophil count, and sera for *Toxoxcara* serological assay by commercial Enzyme-Linked Immunosorbent Assay (ELISA) kit (IBL, International GmBH, Hamburg, Germany). The optical absorbance and OD of all the samples were measured at 450/620 nm and positive, negative and gray zone results were calculated via the kit's cut off. The OD of each sample changed to digits using kit's calculation formula. The numbers from 9–10 are in the range of gray area, numbers less than 10 are negative and more than 10 are positive.

Increasing the IgG titer in patients' sera depends on infection with *Toxocara* or other ascarids. The use of a confirmatory test like Western blot is needed to approve the dependence of IgG on *Toxocara* infection.

We used commercial *Toxocara* western blot IgG kit, LDBIO, Diagnostics, LYON-FRANCE. The specificity of Western blot kit for *T. canis* detection was certified as 100%, and IgG positive sera by ELISA was recon-firmed by Western blot.

All information in this research is confidential and has been left to the researcher and no information has been disclosed elsewhere. Participants were free to enter the study and, after obtaining necessary information about the research, informed consent was taken from the participants.

## Results

**Fig. 1:**
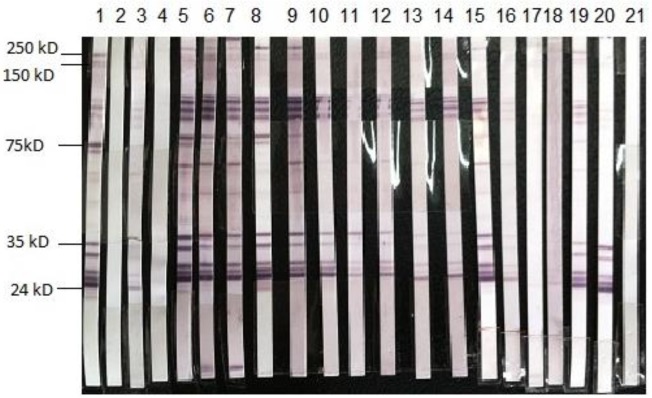
Western blot test which confirm the positive result of ELISA for T*oxoxca canis* antibody, (strip 1 positive control, strip2 negative control, strips 3–21 are confirmed samples except for strips 4,17,18,21

Out of 339 eye patients with uveitis, 229 were male and 110 were female. Totally, 19 cases (5.6%) had a positive ELISA titer for *T. canis* IgG, of which 14 (6.1%) were male and 5 (4.5%) were female, but the control group had a negative ELISA titier for *T. canis* IgG. However, no patient had eosinophilia.The results of the other two variables, sex and contact with dogs, were not statistically significant; however, infection was more frequent in children below 10 yr than other groups (Table 1). Anyway, frequency of anti-*Toxocara* antibody was observed in different types of eye diseases and maximum frequency was seen in the Dacryocystorhinostomy (DCR) group (Table 2). The Western blot test was applied to confirm the presence of specific antibody against *T. canis* in seropositive patients. 15 out of 19 ELISA positive samples were confirmed for *T. canis* infection by 24–35 kDa bands on nitro-cellulose membrane but 4 samples had no band (Fig. 1).

**Table 1:** Epidemiological and demographical factors of eye diseases and control group

***Age(yr)***	***Male***		***Female***		***Total***			
	***Positive***	***Negative***	***Positive***	***Negative***	***Positive***		***Negative***	
	***No***	***No***	***No***	***No***	***No***	***%***	***No***	***%***
<1	0	1	0	0	0	0	1	100
1–10	1	3	0	2	1	16.7	5	83.3
11–20	0	1	0	0	0	0	1	100
21–30	0	2	0	0	0	0	2	100
31–40	1	20	0	11	1	3.12	31	96.8
41–50	4	58	2	35	6	6	93	94
51–60	5	70	2	37	7	6.1	107	93.9
61–70	1	38	0	23	1	1.6	61	98.4
71–80	2	24	1	15	3	7.1	39	92.9
Total	14	217	5	123	19	5.3	340	94.7

**Table 2:** Seroprevalence of antibodies for *Toxocara canis* in the eye diseases and control group

***Type of eye diseases***	***Positive***	***Negative***	***Total***	
	***No***	***No***	***Positive %***	***Negative %***
DCR	7	80	8	92
Glaucoma	0	21	0	100
Cataract	2	58	3.3	96.7
Endophtalmitnis	0	10	0	100
Uveitis	10	151	6.2	93.8
Control	0	20	0	100
Total	19	340	5.3	94.7

## Discussion

Eye diseases have different causes that histological, physiological and congenital disorders, trauma and beside them are some microorganisms. Ophthalmologist usually assessment the agent of eye disorders with ophthalmology instruments, but detection of microbial agents needs different methods. Ocular toxocariasis infection is caused by the second stage larvae of *Toxocara* spp. and is less common in comparison to VLM (9).

In 1977, 41 patients suspected of having clinical *Toxocara* infections were tested by ELISA in the USA, and the results indicated that 38 of them were positive, in which 18 had posterior pole and retinal mass, 3 had both posterior pole and peripheral mass, 12 had peripheral mass, 5 had retinal detachment and 3 had diffuse endophthalmitis, and this confirmed the relationship between eye diseases and toxocariasis (10). In comparison with the present study, their results showed higher prevalence because the samples tested were selected from patients with approved eye diseases, but the present study samples were collected from all types of suspected eye diseases with other agents. Nevertheless, the age similarity in both groups indicates the same prevalence of this infection in younger people, which show relative compatibility.

In another study, 239 patients with clinical manifestations of ocular inflammations, as posterior or peripheral retinochoroiditis, vitritis, papillitis or circumscript endophthalmitis were tested for *Toxocara* infection. Overall, 67 out of 239 patients were seropositive and were divided into two groups: less and more than 14 yr old. All the samples were tested by ELISA and the results were 18.2% and 29.6% seropositive for below and above 14 yr, respectively. The results were confirmed by Western-blot, but are not similar to that of the current study because, in the previous study, the patients were divided into 2 groups: below and above 14 yr, but in the current study, the patients were divided into 5 groups: less than 10 yr, 11–30, 31–50, 51–70 and more than 70 yr (11). The present study result showed that the frequency of seropositive patients in the first group was more than that of other groups and indicated the higher prevalence of toxocariasis in children less than 10 yr, but Logar results showed that people above 14 yr are at higher risk than people below 14 yr and it is vice versa for the present study.

Ocular fluid and aqueous humor of 49 patients were paired in 2 groups: less than (12 persons) and more than 17 yr (37 persons). About 14% of the adults, (5) and 17% of the children (3) were seropositive. All the 3 positive children were male, 2 had posterior uveitis and 1 had pan uveitis (12). In comparison with the present study that divided patients into 4 groups: posterior, anterior, intermediate and pan uveitis, the study shows more prevalence of seropositive results (15%), but both studies showed more seropositivity in children.

The other study on 98 patients with uveitis showed the prevalence of ocular toxocariasis and toxoplasmosis in 2013. The patients consist of 34 anterior uveitis, 39 posterior uveitis and 25 pan uveitis. ELISA, PCR method and eosinophilia count were used for all the blood samples. Finally, Western blot test was conducted for positive samples to confirm the ELISA results.

Eight patients, 6.1% (3 posterior uveitis, 2 pan uveitis and 3 anterior uveitis) were infected by *T. gondii* but 23 patients, 23.5% (4 anterior uveitis, 11 posterior uveitis and 8 pan uveitis) had a positive titer for *T. canis* and all the positive results were reconfirmed by Western blot test. The maximum seropositive results is related to male posterior uveitis patients 47%, which is similar to the results of the present study (13). The other study considered the trend of toxocariasis researches in Iran, this study was performed to analyze different features of the *Toxocara* especially clinical sings in patients, new diagnostic tools, especially rapid diagnostic tests, and new treatment technique for future projects (4). The last report about eye toxocariasis was from the authors of this manuscript from different ophthalmology hospitals of Tehran. They indicated the relation between toxocariasis and different type of uveitis against age and contact with dog and other risk factors by ELISA and western blot (5).

However, some different case reports have shown the presence of ocular toxocariasis in different parts of Iran, as the reports on ocular toxocariasis from Babol City. A 40 yr old man with vision disorders and clinical symptoms in the eye were considered to have Toxocariasis by IFA test. A high IgG titer of *Toxoxcara* and ocular vision disorders confirmed the patient's Toxocariasis (14). Another report on a 29-yr-old man with a history of decreased vision and visual acuity in his left eye with anterior vitreous was considered. Serologic test (ELISA) for toxocariasis was strongly positive (15). Another report was on an 11-year-old girl with bilateral vision disorders, referred to Nikoukari Tabriz hospital. After ophthalmology consideration and serologic test, the patient with high antibody titer (ELISA) was infected by *Toxocara* larvae (16). In addition to all these reports, another researcher reported a *T. cati* larvae, from the eye of a child. All these reports confirmed the present study's results that Toxocariasis was seen in different age and gender, and it is the cause of some eye disease (17).

## Conclusion

Ocular toxocariasis is prevalent and children are more likely to be at risk of the infection than other age groups. Using ELISA + Western-blot is a reliable method for detecting toxocariasis. All the patients with inflammatory eye diseases such as endophthalmitis, uveitis DCR, glaucoma and cataract were studied for *Toxocara* infection in this research work were at risk. Therefore, in contrast to the previous idea, all eye inflammatory diseases in ocular patients should be considered for *Toxocara* antibodies in addition to Uveitis.
